# Effects of immunosuppressive treatment on microsomal prostaglandin E synthase 1 and cyclooxygenases expression in muscle tissue of patients with polymyositis or dermatomyositis

**DOI:** 10.1136/ard.2007.079525

**Published:** 2007-12-18

**Authors:** M Korotkova, S Barbasso Helmers, I Loell, H Alexanderson, C Grundtman, C Dorph, I E Lundberg, P-J Jakobsson

**Affiliations:** 1 Department of Medicine, Rheumatology Unit, Karolinska Institutet/Karolinska University Hospital, Solna, Stockholm, Sweden; 2 Department of Medicine, Rheumatology Unit and Karolinska Biomic Center, Karolinska Institutet, Stockholm, Sweden

## Abstract

**Objectives:**

To investigate the expression of microsomal prostaglandin E (PGE) synthase 1 (mPGES-1) and cyclooxygenase (COX) in muscle biopsies from patients with polymyositis or dermatomyositis before and after conventional immunosuppressive treatment.

**Methods:**

mPGES-1 and COX expression was evaluated by immunohistochemistry in muscle tissue from healthy individuals and from patients with polymyositis or dermatomyositis before and after conventional immunosuppressive treatment. The number of inflammatory cell infiltrates, T lymphocytes and macrophages was estimated before and after treatment. To localise the mPGES-1 expression double immunofluorescence was performed with antibodies against mPGES-1, CD3, CD68, CD163 and a fibroblast marker. A functional index was used to assess muscle function.

**Results:**

In patients with myositis, mPGES-1, COX-2 and COX-1 expression was significantly higher compared to healthy individuals and associated with inflammatory cells. Double immunofluorescence demonstrated a predominant expression of mPGES-1 in macrophages. Conventional immunosuppressive treatment resulted in improved but still lower muscle function than normal. A decreased number of CD68-positive macrophages and reduced COX-2 expression in muscle tissue was also seen. By contrast, following the same treatment no significant changes were observed in muscle tissue regarding number of infiltrates, T lymphocytes, CD163-positive macrophages or mPGES-1 protein levels.

**Conclusions:**

Increased expression of mPGES-1, COX-1 and COX-2 at protein level was observed in muscle tissue from patients with myositis compared to healthy individuals. Conventional immunosuppressive treatment led to a significant downregulation of COX-2 in myositis muscle tissue. However, the expression of mPGES-1 and COX-1 remained unchanged indicating a role of these enzymes in the chronicity of these diseases.

Polymyositis and dermatomyositis are chronic muscle disorders characterised by muscle weakness and fatigue and by skin involvement in the case of dermatomyositis.^1^ These diseases are also characterised by infiltration of inflammatory cells in skeletal muscle tissue, muscle fibre degeneration and regeneration. The pathogenesis of myositis has not been well characterised yet, but pro-inflammatory cytokines have been consistently found in the inflamed muscle and are implicated in the pathogenesis.^2^^–^4

Arachidonic acid metabolites such as prostaglandins (PG) might also contribute to the pathogenesis of inflammatory myositis. Human skeletal muscles have a considerable capacity to produce PGE_2_, PGD_2_, PGF_2α_ and PGI_2_.^5^ PGE_2_ appears to be involved in a number of biological processes, including protein turnover and myogenesis, and is a potent mediator of muscular pain and inflammation.^6^^–^10 Interleukin (IL)1β and tumour necrosis factor (TNF), which are markedly expressed in myositis muscle tissue, stimulate PGE_2_ production in skeletal muscles.^2^ ^3^ ^11^ ^12^ In the PGE_2_ biosynthetic pathway, cyclooxygenase (COX)-1 and COX-2 catalyse the conversion of arachidonic acid into PGH_2_ (fig 1). Recently, enhanced expression of COX-1 and COX-2 mRNA was demonstrated in inflamed muscle tissue from patients with myositis, suggesting a role for them in this disease.^13^ Three terminal PGE synthases (PGES) catalyse the formation of PGE_2_ from PGH_2_ Microsomal PGE synthase 1 (mPGES-1) is strongly induced by proinflammatory stimuli in various cells and preferentially couples with COX-2 contributing to significant PGE_2_ release.^14^^–^16 Cytosolic PGES (cPGES) and mPGES-2 are constitutively expressed and likely to function in the basal production of PGE_2_^17^ ^18^ Studies of mPGES-1 –/– knock-out mice have demonstrated a critical role for mPGES-1 in the development of pain, fever and inflammation.^19^ ^20^ mPGES-1 is upregulated in a range of inflammatory diseases and considered a new target for therapeutic strategies to control induced PGE_2_ synthesis.^21^^–^23 However, the expression of mPGES-1 in muscle tissue from patients with myositis has not been studied.

**Figure 1:**
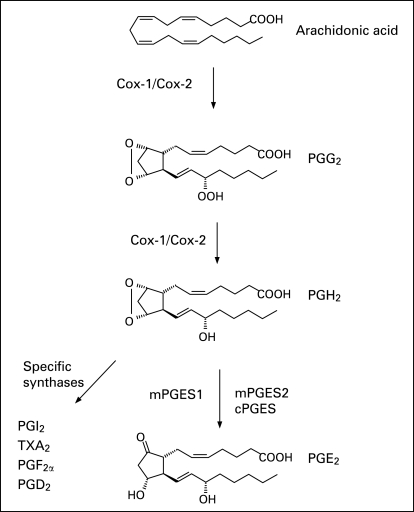
A schematic overview of the prostaglandin (PG) biosynthesis cascade.

Despite clinical improvement with conventional immunosuppressive treatment including high doses of glucocorticoids (GC), many patients with myositis experience persistent muscle weakness. There are also reports of persisting inflammatory cells and increased expression of IL1 in muscle tissue despite long-time treatment with high doses of GC.^3^ ^24^ Intra-articular treatment with GC significantly reduces mPGES-1, COX-1 and COX-2 expression in the synovial tissue from patients with rheumatoid arthritis and is associated with clinical improvement.^25^ Whether mPGES-1 and COX expression in skeletal muscle from patients with myositis is affected by immunosuppressive treatment has not been investigated to date.

In the present study we examined the expression and localisation of mPGES-1 and COX in skeletal muscle tissue from patients with polymyositis or dermatomyositis and from healthy subjects. In addition we studied the effects of immunosuppressive treatment on mPGES-1 and COX expression in skeletal muscle tissue from patients with myositis in relation to the effects on clinical function.

## PATIENTS AND METHODS

### Patients and muscle biopsies

In the first cohort, nine patients with recently diagnosed polymyositis or dermatomyositis and three with treatment-resistant myositis (disease duration 3–6 years) meeting the Bohan and Peter criteria were included (median age 54 years, range 44–76 years).^26^ ^27^ Clinical data of the patients are presented in tables 1 and 2. Detailed clinical data for these patients have been reported previously.^28^

**Table 1:** Clinical data on the patients at the time of biopsies, cohort 1: patients with recently diagnosed and with treatment-resistant myositis (marked with *)

Patient	Age	Sex	Bohan and Peter diagnostic criteria	Biopsy site	Treatment at time of biopsy, mg/day
1	53	F	Probable polymyositis	Mus vastus lat	None
2	59	F	Probable polymyositis	Mus deltoideus	Pred 5, NSAID
3	76	F	Definite polymyositis	Mus vastus lat	None
4	44	F	Definite polymyositis	Mus vastus lat	None
5	65	F	Definite dermatomyositis	Mus vastus lat	Pred 15
6	72	F	Definite polymyositis	Mus vastus lat	Pred 15–40, AZA
7	60	M	Probable polymyositis	Mus vastus lat	NSAID
8	56	M	Definite polymyositis	Mus vastus lat	NSAID
9	55	M	Probable dermatomyositis	Mus vastus lat	None
10*	67	M	Definite dermatomyositis	Mus vastus lat	AZA, MTX, Cs
11*	54	F	Probable polymyositis	Mus tib ant	AZA, MTX, Cs
12*	61	F	Definite polymyositis	Mus vastus lat	AZA, MTX, Cs

Ant, anterior; AZA, azathioprine; Cs, ciclosporine; F, female; lat, lateralis; M, male; Mus, musculus; MTX, methotrexate; NSAID, non-steroidal anti-inflammatory drugs; tib, tibialis; Pred, prednisolone.

**Table 2:** Clinical data on the patients at the time of biopsies, cohort 2: myositis patients before and after conventional immunosuppressive treatment

Patient	Age	Sex	Bohan and Peter diagnostic criteria	Treatment at time of biopsy 1, mg/day	Treatment at time of biopsy 2, mg/day
1	60	F	Definite polymyositis	None	Pred 12.5, MTX
2	23	F	Probable dermatomyositis	None	Pred 5, AZA
3	49	F	Definite dermatomyositis	NSAID	Pred 15, NSAID
4	53	F	Definite polymyositis	None	Pred 20, AZA
5	41	F	Probable dermatomyositis	None	Pred 30
6	44	F	Probable dermatomyositis	None	Pred 7.5
7	67	M	Probable polymyositis	None	Pred 10
8	88	F	Definite dermatomyositis	None	Pred 7.5
9	60	M	Probable polymyositis	NSAID	Pred 30
10	62	M	Definite dermatomyositis	None	Pred 10, AZA

AZA, azathioprine; F, female; M, male; MTX, methotrexate; NSAID, non-steroidal anti-inflammatory drugs; Pred, prednisolone.

To investigate the effects of immunosuppressive treatment a second cohort of 10 patients with recently diagnosed polymyositis or dermatomyositis with available follow-up biopsies was included (median age 56.5 years, range 23–88 years). The patients were initially treated with oral prednisolone (40–60 mg/day) with slowly tapering doses and all patients (except two) received an additional immunosuppressive agent (tables 1 and 2). Two patients were treated with non-steroidal anti-inflammatory drugs (NSAIDs) at the time of the first biopsy. Muscle tissue biopsies were taken from m. vastus lateralis before and after a median of 8.5 months (range 4–11 months) with immunosuppressive treatment. Muscle tissue biopsies were obtained under local anaesthesia using the semi-open biopsy technique.^28^ ^29^

Muscle biopsies from seven healthy individuals (four women and three men, median age 46 years, range 38–50 years) without clinical or histopathological signs of muscle disease were included as controls. Muscle biopsies were obtained from musculus vastus lateralis in six individuals and from musculus tibialis anterior in one individual.

The approval was granted by the Ethics Committee at the Karolinska University Hospital, Stockholm and all patients and controls gave their informed consent to participate in the study.

### Clinical assessment of patients

Muscle performance (functional index (FI) of myositis) was assessed by the number of repetitions performed in defined muscle groups before treatment and at the time of the second biopsy.^30^ The individual total score is presented as percentage of the maximal score 64 (mean values of left and right side). The responder criterion was set to 20% improvement.

### Immunohistochemical analysis

Staining of serial cryostat sections with mouse monoclonal anti-CD3 (BD Biosciences, San José, California, USA), anti-CD68 (marker of monocyte/macrophage lineage, KP-1 clone, Dako Cytomation, Glostrup, Denmark) and anti-CD163 (resident tissue macrophage marker, BerMac3 clone, Dako Cytomation) antibodies was performed using a standard protocol.^31^ Staining with rabbit polyclonal anti-human mPGES-1 antiserum,^22^ polyclonal anti-cPGES, anti-COX-2 and anti-COX-1 (Cayman Chemicals, Ann Arbor, Michigan, USA) and mouse monoclonal anti-COX-1 antibodies (Wako Chemicals, Neuss, Germany) was performed as previously described.^32^ Staining in skeletal muscle tissue was halted by preincubation of anti-mPGES-1 serum with mPGES-1 protein and by preincubation of commercial antibodies with respective blocking peptides (Cayman Chemical). Isotype-matched irrelevant antibodies were used as negative controls. The first and the last sections from each series of consecutive sections were stained with haematoxylin and eosin to evaluate the number of inflammatory infiltrates.

Stained tissue sections were examined using a Polyvar II microscope (Reichert-Jung, Vienna, Austria) and photographed with a digital Leica camera 300F (Leica, Cambridge, UK). The number of PGES and COX positive cells was assessed by conventional microscopy measurements of the entire tissue section (2–9 mm^2^) using semi-quantitative scale: 0, no staining; 1, a few stained scattered cells; 2, many stained scattered cells; 3, many stained scattered cells and cells in one infiltrate; 4, strong staining in many scattered cells and several infiltrates. Evaluation of coded sections was performed by two independent observers. The mean scores from the two assessments were used for statistical analysis.

Expression of CD3, CD68 and CD163 was assessed quantitatively using computer-assisted image analysis. The images were analysed with a Quantimet 600 image analyser (Leica) and positive staining was expressed as percentage of total counterstained tissue area.

Double immunofluorescence was performed using anti-human mPGES-1 antiserum and mouse monoclonal anti-CD3, anti-CD68, anti-CD163 and anti-prolyl-4-hydroxylase (fibroblast marker, 5B5 clone, Dako Cytomation) antibodies as published previously.^22^

### Statistical analysis

Data were analysed using the Mann–Whitney U test and Wilcoxon signed rank test and Bonferroni corrections for multiple comparisons. p Values <0.05 were considered statistically significant. Correlation between muscle FI and enzyme expression in muscle tissue was analysed using Spearman rank correlation test.

## RESULTS

### Expression of PGES and COX

**Figure 2:**
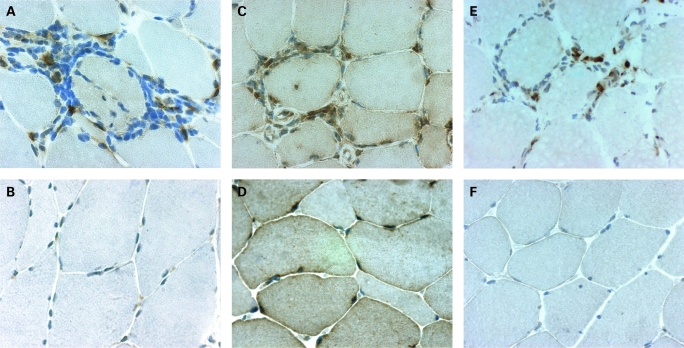
Immunohistochemical staining (brown) for (A, B) microsomal prostaglandin E synthase (mPGES)-1, (C, D) cyclooxygenase (COX)-1 and (E, F) COX-2 in representative muscle tissue sections counterstained with haematoxylin (A, C, E) from patients with polymyositis and (B, D, F) from healthy individuals (original magnification ×500).

**Figure 3:**
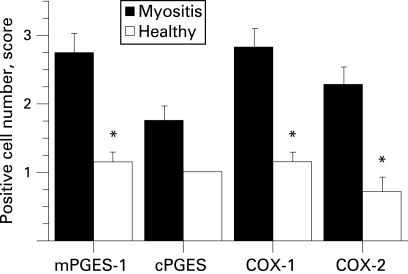
Expression of microsomal prostaglandin E synthase (mPGES)-1, cytosolic PGES (cPGES), cyclooxygenase (COX)-1 and COX-2 in muscle tissue from patients with polymyositis or dermatomyositis and from healthy individuals. Results are expressed as positive cell score mean (standard error of mean (SEM)). *p<0.05, patients vs healthy individuals.

A marked mPGES-1 staining localised in many scattered mononuclear cells and in mononuclear cells in infiltrates was observed in all patients with some interindividual variations (fig 2A). For 6 patients mPGES-1 staining was additionally localised to smooth muscle cells in large vessels, and for 10 patients to capillaries as well. In muscle tissue from healthy individuals, weak mPGES-1 staining was detected in a few scattered mononuclear cells and capillaries (fig 2B), as well as in smooth muscle cells in large vessels in two individuals. Using conventional microscopic assessment, we found that mPGES-1 expression in muscle tissue from patients with myositis (n = 12) was significantly higher (p<0.01) when compared to healthy individual tissue (n = 7) (fig 3).

Staining of cPGES was observed in scattered mononuclear cells, cells surrounding large vessels and muscle fibres in myositis and healthy muscle tissues with a similar distribution pattern (data not shown). In two patient biopsies, cPGES positive cells were detected in inflammatory cells in infiltrates. The staining for cPGES in the myositis muscle was not significantly different when compared to healthy controls (fig 3).

COX-1 expression was detected in muscle fibres, blood vessels and scattered mononuclear cells in patients and healthy subjects. In patients a strong COX-1 staining was additionally observed in mononuclear cells within the inflammatory infiltrates (fig 2C, D). COX-2 was expressed in macrophage-like cells within inflammatory infiltrates, in scattered mononuclear cells and in some large vessels in myositis muscle tissue. By contrast, in healthy muscle tissue COX-2 staining was only detected in few scattered mononuclear cells and in some vessels (fig 2E, F). COX-1 and COX-2 expression were significantly enhanced (p<0.01) in myositis muscle tissue when compared to healthy control (fig 3).

**Figure 4:**
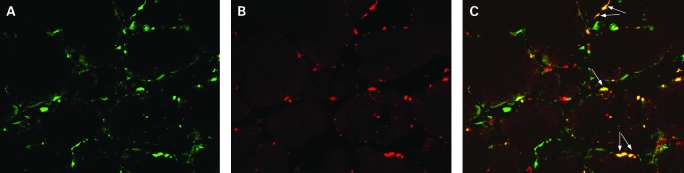
Double fluorescence staining demonstrating cellular localisation of microsomal prostaglandin E synthase (mPGES)-1: (A) mPGES-1 positive (green) cells, (B) CD163 positive (red) cells and (C) double stained (yellow) cells in representative muscle tissue section from the patient with polymyositis (original magnification ×250).

Double immunofluorescence revealed the expression of mPGES-1 in CD163-positive (fig 4A–C) and of CD68-positive macrophages (data not shown). However, we could not detect any mPGES-1 staining in T lymphocytes or fibroblasts (data not shown).

### Effects of immunosuppressive treatment on PGES and COX expression

**Figure 5:**
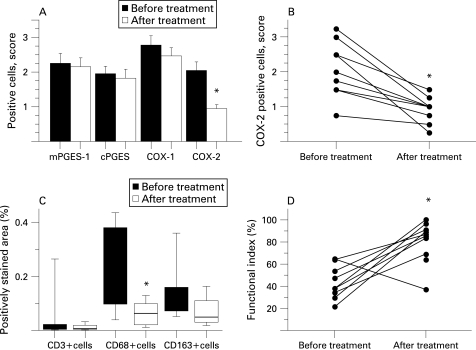
A. Expression of microsomal prostaglandin E synthase (mPGES)-1 and related enzymes in muscle tissues of patients with polymyositis or dermatomyositis before and after conventional treatment. Results are expressed as positive cell score mean (standard error of mean (SEM)). B. Expression of cyclooxygenase (COX)-2 in muscle tissues of patients with polymyositis or dermatomyositis before and after conventional treatment. Results are expressed as positive cell score. C. Number of inflammatory cells in muscle tissue from patients with polymyositis and dermatomyositis before and after conventional treatment. Results are expressed as percentage of the total area of counterstained tissue. D. Effects of conventional treatment on muscle function in patients with polymyositis or dermatomyositis. Results are expressed as percentage of functional index (FI) for each patient. *p<0.05, before vs after treatment.

The expression of mPGES-1 in myositis muscle tissue was not altered by the treatment (n = 10) (fig 5A). In patients without infiltrates after treatment (4 out of 10), mPGES-1 staining was still apparent in scattered mononuclear cells. Likewise, the distribution pattern or score for cPGES and COX-1 positive cells remained unchanged (fig 5A). By contrast, the score for COX-2 positive cells in muscle tissue was significantly reduced (p<0.01) after treatment (fig 5A, B). There was no significant difference between polymyositis and dermatomyositis regarding the expression pattern of these enzymes before or after treatment.

### Effects of immunosuppressive treatment on muscle histopathology

Before treatment, 8 out of 10 patients presented infiltrates of mononuclear cells. After 4–11 months of immunosuppressive treatment the number of inflammatory infiltrates tended to be lower (median 0.52, range 0–2.6 infiltrates/mm^2^ vs median 0.22, range 0–2.4 infiltrates/mm^2^ before and after treatment, respectively), but this difference did not reach statistical significance.

Positive staining for CD3, CD68 and CD163 markers was detected in infiltrates, in scattered cells and in cells surrounding large vessels. The total number of monocytes/macrophages (CD68 positive cells) was significantly reduced after the treatment (p<0.01), while the positively stained area was not altered for T lymphocytes or CD163-positive resident macrophages (fig 5C).

### Effects of immunosuppressive treatment on muscle function

Before treatment, muscle weakness in patients was confirmed by a reduced FI (median 38.4%, range 22–65%), performed in all but one patient (n = 9). After 4–11 months of immunosuppressive treatment, a significantly increased FI was recorded (median 86.3%, range 37.5–100%, p<0.05), reflecting improved muscle function (fig 5D). On an individual basis, 8 out of 10 patients had improved by more than 20% in FI score. For one patient a decreased FI score was noted and for another the results of the first FI was not available. Despite the marked improvement in muscle function during the treatment period, a majority of the patients still had FI scores below the maximal value. Only one patient improved up to maximal score (100%).

There was no correlation between enzyme expression in muscle tissue and FI before or after treatment and the changes in enzyme expression did not correlate with changes in FI after treatment. In the patient who deteriorated clinically, the score for COX-2 expression was decreased after the treatment (from 2.5 to 1.5), in similar fashion to other patients who clinically improved.

## DISCUSSION

The present study demonstrates significantly increased expression of mPGES-1, COX-2 and COX-1 in skeletal muscle tissue from patients with polymyositis or dermatomyositis compared to that from healthy individuals. In healthy muscle tissue COX-1 and cPGES proteins were detected in scattered mononuclear cells, in vessels and in muscle fibres, suggesting that these enzymes account for the basal production of PGE_2_. In healthy muscle tissue we also observed mPGES-1 and COX-2 staining in few scattered mononuclear cells and blood vessels, indicating constitutive expression of these enzymes in muscle tissue. Constitutive expression of mPGES-1 and COX-2 has been reported in other tissues, eg, kidney, suggesting their possible role for basal PGE_2_ production under non-pathological conditions.^33^ ^34^ Moreover, increased expression of COX-2 and mPGES-1 has been demonstrated in response to non-inflammatory stimuli, such as mechanical stretch and mechanical stress in certain cells.^8^ ^35^ ^36^

The observed enhanced expression of COX-1 and COX-2 in muscle tissue from patients with myositis is in accordance with previously published data demonstrating increased levels of COX-1 and COX-2 mRNA in inflammatory cells and vessels in muscle tissue from patients with myositis.^13^ Notably there have been no clinical trials addressing specifically the impact of selective or non-selective NSAIDs in patients with myositis. In addition, mPGES-1 expression was enhanced in myositis muscle tissue, as an increased number of positively stained scattered mononuclear cells and as positively stained cells within inflammatory infiltrates compared to low constitutive expression in healthy muscle tissue. Using double staining, we identified CD68 and CD163-positive macrophages as the major cell types expressing mPGES-1. IL1β and TNF are strongly expressed in myositis skeletal muscle tissue^2^ ^3^ and known to maintain mPGES-1 expression in macrophages, consequently contributing to the enhanced release of PGE_2_ and inflammation in the muscle tissue.

We also examined the effects of conventional immunosuppressive treatment on muscle functional activity, muscle histopathology and expression of mPGES-1/COX in muscle tissues. Treatment resulted in significant improved muscle function in the majority of the patients. However, most patients still had impaired muscle function at the time of the second biopsy. Significant reduction in the total number of macrophages (CD68 positive cells) confirmed anti-inflammatory effects of the treatment. After treatment the COX-2 positive cells in muscle tissue were significantly decreased, while the expression of mPGES-1, cPGES or COX-1 was not suppressed. Downregulation of COX-2 expression is one of the expected anti-inflammatory effects of GC and is associated with suppression of PGE_2_ biosynthesis.

However, there was no correlation between COX-2 expression in muscle tissue and FI before or after treatment, probably due to the small number of observations.

The reduced number of COX-2 positive cells could be explained by GC dependent downregulation of COX-2 expression and/or a significant reduction in total number of macrophages (CD68 positive cells). By contrast, resident tissue macrophages (CD163-positive cells) did not decrease significantly after treatment. It is tempting to speculate that the population of CD68 cells that decreased as a result of the treatment constitutes cells that did not express CD163 or mPGES-1, as these molecules were not changed after treatment. A similar relative persistence of synovial CD163-positive resident tissue macrophages compared to infiltrating macrophages during anti-TNF treatment has been demonstrated in chronic autoimmune arthritis.^37^ In addition, immunosuppressive treatment did not affect the number of T lymphocytes in myositis muscle providing a basis for persisting immune reaction that targets muscle fibres.

Interestingly, in some conditions mPGES-1 functionally utilises PGH_2_ generated by COX-1.^34^ ^38^ ^39^ The persisting expression of mPGES-1 and COX-1 in inflamed muscle tissue despite treatment might preserve PGE_2_ production and contribute to chronic muscle inflammation. However, the role of the mPGES-1/COX-1 pathway in overall PGE_2_ production in muscle tissue remains to be elucidated.

Recent data suggest that COX-2–dependent PG synthesis is important for skeletal muscle regeneration.^40^^–^42 While COX-2 inhibition reduces inflammation to a large extent due to the suppression of PGE_2_ formation, it might impede the functional recovery of muscles via the suppression of other PGs. In this context mPGES-1 may constitute a more selective and safe therapeutic target than COX-2. Selective inhibition of mPGES-1 will allow for intact baseline PGE_2_ production as well as intact production of other PG important for muscle regeneration and, ultimately, constitute a more preferable anti-inflammatory treatment than the currently used systemic GCs.

In conclusion, we have demonstrated a significantly enhanced expression of mPGES-1, COX-2 and COX-1 in patients with polymyositis or dermatomyositis compared to healthy controls, suggesting its role in the pathogenesis of these diseases. Moreover, we have shown for the first time that conventional immunosuppressive treatment led to a significant downregulation of COX-2 in myositis muscle tissue, while the expression of mPGES-1 and COX-1 remained unchanged. This persisting expression of mPGES-1 and COX-1 may have a role in the chronicity of myositis.

## References

[1] Mastaglia FL Garlepp MJ Phillips BA Zilko PJ Inflammatory myopathies: clinical, diagnostic and therapeutic aspects. Muscle Nerve 2003;27:407–25 12661042 10.1002/mus.10313

[2] Lundberg I Ulfgren AK Nyberg P Andersson U Klareskog L Cytokine production in muscle tissue of patients with idiopathic inflammatory myopathies. Arthritis Rheum 1997;40:865–74 9153548 10.1002/art.1780400514

[3] Nyberg P Wikman AL Nennesmo I Lundberg I Increased expression of interleukin 1α and MHC class I in muscle tissue of patients with chronic, inactive polymyositis and dermatomyositis. J Rheumatol 2000;27:940–8 10782820

[4] Figarella-Branger D Civatte M Bartoli C Pellissier JF Cytokines, chemokines, and cell adhesion molecules in inflammatory myopathies. Muscle Nerve 2003;28:659–82 14639580 10.1002/mus.10462

[5] Berlin T Cronestrand R Nowak J Sonnenfeld T Wennmalm A Conversion of arachidonic acid to prostaglandins in homogenates of human skeletal muscle and kidney. Acta Physiol Scand 1979;106:441–5 495152 10.1111/j.1748-1716.1979.tb06424.x

[6] Rodemann HP Goldberg AL Arachidonic acid, prostaglandin E2 and F2 α influence rates of protein turnover in skeletal and cardiac muscle. J Biol Chem 1982;257:1632–8 6799511

[7] Zalin RJ The role of hormones and prostanoids in the in vitro proliferation and differentiation of human myoblasts. Exp Cell Res 1987;172:265–81 3308494 10.1016/0014-4827(87)90386-7

[8] Otis JS Burkholder TJ Pavlath GK Stretch-induced myoblast proliferation is dependent on the COX2 pathway. Exp Cell Res 2005;310:417–25 16168411 10.1016/j.yexcr.2005.08.009

[9] Hedenberg-Agnusson B Ernberg M Alstergren P Kopp S Pain mediation by prostaglandin E2 and leukotriene B4 in the human masseter muscle. Acta Odontol Scand 2001;59:348–55 11831483 10.1080/000163501317153185

[10] Tegeder L Zimmermann J Meller ST Geisslinger G Release of algesic substances in human experimental muscle pain. Inflamm Res 2002;51:393–402 12234056 10.1007/pl00000320

[11] Baracos V Rodemann HP Dinarello CA Goldberg AL Stimulation of muscle protein degradation and prostaglandin E2 release by leukocytic pyrogen (interleukin-1). A mechanism for the increased degradation of muscle proteins during fever. N Engl J Med 1983;308:553–8 6402699 10.1056/NEJM198303103081002

[12] Schafers M Sorkin LS Sommer C Intramuscular injection of tumor necrosis factor-α induces muscle hyperalgesia in rats. Pain 2003;104:579–88 12927630 10.1016/S0304-3959(03)00115-5

[13] Studynkova JT Kuchen S Jeisy E Schedel J Charvat F Jarosova K The expression of cyclooxygenase-1, cyclooxygenase-2 and 5-lipoxygenase in inflammatory muscle tissue of patients with polymyositis and dermatomyositis. Clin Exp Rheumatol 2004;22:395–402 15301234

[14] Jakobsson PJ Thoren S Morgenstern R Samuelsson B Identification of human prostaglandin E synthase: a microsomal, glutathione-dependent, inducible enzyme, constituting a potential novel drug target. Proc Natl Acad Sci USA 1999;96:7220–5 10377395 10.1073/pnas.96.13.7220 PMC22058

[15] Thoren S Weinander R Saha S Jegerschold C Pettersson PL Samuelsson B Human microsomal prostaglandin E synthase-1: purification, functional characterization and projection structure determination. J Biol Chem 2003;278:22199–209 12672824 10.1074/jbc.M303227200

[16] Murakami M Naraba H Tanioka T Semmyo N Nakatani Y Kojima F Regulation of prostaglandin E2 biosynthesis by inducible membrane-associated prostaglandin E2 synthase that acts in concert with cyclooxygenase-2. J Biol Chem 2000;275:32783–92 10869354 10.1074/jbc.M003505200

[17] Murakami M Nakashima K Kamei D Masuda S Ishikawa Y Ishii T Cellular prostaglandin E2 production by membrane-bound prostaglandin E synthase-2 via both cyclooxygenases-1 and -2. J Biol Chem 2003;278:37937–47 12835322 10.1074/jbc.M305108200

[18] Tanioka T Nakatani Y Semmyo N Murakami M Kudo I Molecular identification of cytosolic prostaglandin E2 synthase that is functionally coupled with cyclooxygenase-1 in immediate prostaglandin E2 biosynthesis. J Biol Chem 2000;275:32775–82 10922363 10.1074/jbc.M003504200

[19] Engblom D Saha S Engstrom L Westman M Audoly LP Jakobsson PJ Microsomal prostaglandin E synthase-1 is the central switch during immune-induced pyresis. Nat Neurosci 2003;6:1137–8 14566340 10.1038/nn1137

[20] Trebino CE Stock JL Gibbons CP Naiman BM Wachtmann TS Umland JP Impaired inflammatory and pain responses in mice lacking an inducible prostaglandin E synthase. Proc Natl Acad Sci USA 2003;100:9044–9 12835414 10.1073/pnas.1332766100 PMC166435

[21] Subbaramaiah K Yoshimatsu K Scherl E Das KM Glazier KD Golijanin D Microsomal prostaglandin E synthase-1 is overexpressed in inflammatory bowel disease. Evidence for involvement of the transcription factor Egr-1. J Biol Chem 2004;279:12647–58 14722058 10.1074/jbc.M312972200

[22] Westman M Korotkova M af Klint E Stark A Audoly LP Klareskog L Expression of microsomal prostaglandin E synthase 1 in rheumatoid arthritis synovium. Arthritis Rheum 2004;50:1774–80 15188353 10.1002/art.20286

[23] Cipollone F Fazia M Iezzi A Ciabattoni G Pini B Cuccurullo C Balance between PGD synthase and PGE synthase is a major determinant of atherosclerotic plaque instability in humans. Arterioscler Thromb Vasc Biol 2004;24:1259–65 15155382 10.1161/01.ATV.0000133192.39901.be

[24] Lundberg I Kratz AK Alexanderson H Patarroyo M Decreased expression of interleukin-1α, interleukin-1β, and cell adhesion molecules in muscle tissue following corticosteroid treatment in patients with polymyositis and dermatomyositis. Arthritis Rheum 2000;43:336–48 10693873 10.1002/1529-0131(200002)43:2<336::AID-ANR13>3.0.CO;2-V

[25] Korotkova M Westman M Gheorghe KR af Klint E Trollmo C Ulfgren AK Effects of antirheumatic treatments on the prostaglandin E2 biosynthetic pathway. Arthritis Rheum 2005;52:3439–47 16255020 10.1002/art.21390

[26] Bohan A Peter JB Polymyositis and dermatomyositis (part 1). N Engl J Med 1975;292:344–7 1090839 10.1056/NEJM197502132920706

[27] Bohan A Peter JB Polymyositis and dermatomyositis (part 2). N Engl J Med 1975;292:403–7 1089199 10.1056/NEJM197502202920807

[28] Dorph C Englund P Nennesmo I Lundberg IE Signs of inflammation in both symptomatic and asymptomatic muscles from patients with polymyositis and dermatomyositis. Ann Rheum Dis 2006;65:1565–71 16831829 10.1136/ard.2005.051086 PMC1798445

[29] Henriksson KG “Semi-open” muscle biopsy technique. A simple outpatient procedure. Acta Neurol Scand 1979;59:317–23 484204

[30] Alexanderson H Stenstrom CH Lundberg I Safety of a home exercise programme in patients with polymyositis and dermatomyositis: a pilot study. Rheumatology (Oxford) 1999;38:608–11 10461472 10.1093/rheumatology/38.7.608

[31] Frostegard J Ulfgren AK Nyberg P Hedin U Swedenborg J Andersson U Cytokine expression in advanced human atherosclerotic plaques: dominance of pro-inflammatory (Th1) and macrophage-stimulating cytokines. Atherosclerosis 1999;145:33–43 10428293 10.1016/s0021-9150(99)00011-8

[32] Ulfgren AK Lindblad S Klareskog L Andersson J Andersson U Detection of cytokine producing cells in the synovial membrane from patients with rheumatoid arthritis. Ann Rheum Dis 1995;54:654–61 7677442 10.1136/ard.54.8.654 PMC1009963

[33] Schneider A Zhang Y Zhang M Lu WJ Rao R Fan X Membrane-associated PGE synthase-1 (mPGES-1) is coexpressed with both COX-1 and COX-2 in the kidney. Kidney Int 2004;65:1205–13 15086459 10.1111/j.1523-1755.2004.00493.x

[34] Boulet L Ouellet M Bateman KP Ethier D Percival MD Riendeau D Deletion of microsomal prostaglandin E2 (PGE2) synthase-1 reduces inducible and basal PGE2 production and alters the gastric prostanoid profile. J Biol Chem 2004;279:23229–37 15016822 10.1074/jbc.M400443200

[35] Park JM Yang T Arend LJ Schnermann JB Peters CA Freeman MR Obstruction stimulates COX-2 expression in bladder smooth muscle cells via increased mechanical stretch. Am J Physiol 1999;276:F129–36 9887088 10.1152/ajprenal.1999.276.1.F129

[36] Gosset M Berenbaum F Levy A Pigenet A Thirion S Saffar JL Prostaglandin E2 synthesis in cartilage explants under compression: mPGES-1 is a mechanosensitive gene. Arthritis Res Ther 2006;8:R135 16872525 10.1186/ar2024 PMC1779392

[37] De Rycke L Baeten D Foell D Kruithof E Veys EM Roth J Differential expression and response to anti-TNFα treatment of infiltrating versus resident tissue macrophage subsets in autoimmune arthritis. J Pathol 2005;206:17–27 15809977 10.1002/path.1758

[38] Dieter P Scheibe R Jakobsson PJ Watanabe K Kolada A Kamionka S Functional coupling of cyclooxygenase 1 and 2 to discrete prostanoid synthases in liver macrophages. Biochem Biophys Res Commun 2000;276:488–92 11027502 10.1006/bbrc.2000.3496

[39] Bezugla Y Kolada A Kamionka S Bernard B Scheibe R Dieter P COX-1 and COX-2 contribute differentially to the LPS-induced release of PGE2 and TxA2 in liver macrophages. Prostaglandins Other Lipid Mediat 2006;79:93–100 16516813 10.1016/j.prostaglandins.2005.11.001

[40] Mendias CL Tatsumi R Allen RE Role of cyclooxygenase-1 and -2 in satellite cell proliferation, differentiation, and fusion. Muscle Nerve 2004;30:497–500 15372441 10.1002/mus.20102

[41] Shen W Prisk VR Li Y Foster W Huard J Inhibited skeletal muscle healing in cyclooxygenase-2 gene-deficient mice: the role of PGE2 and PGF2α. J Appl Physiol 2006;101:1215–21 16778000 10.1152/japplphysiol.01331.2005

[42] Bondesen BA Mills ST Pavlath GK The COX-2 pathway regulates growth of atrophied muscle via multiple mechanisms. Am J Physiol Cell Physiol 2006;290:C1651–9 16467402 10.1152/ajpcell.00518.2005

